# An AHP based approach to forecast groundwater level at potential recharge zones of Uckermark District, Brandenburg, Germany

**DOI:** 10.1038/s41598-022-10403-9

**Published:** 2022-04-16

**Authors:** Ahmed Tahmid Raihan, Sonja Bauer, Sayan Mukhopadhaya

**Affiliations:** 1 Hochschule für Technik Stuttgart, Stuttgart, Germany; 2 BASF Digital Farming GmbH, Köln, Germany

**Keywords:** Environmental impact, Hydrology

## Abstract

Uckermark, a district of the state Brandenburg, Germany is situated in one of the driest regions of Germany. The district is known for its agricultural activities and natural resources. But in recent times the district is being prone to groundwater deficit due to the dryness of its climate. In this research initiative, a GIS and Remote Sensing based approach has been made to detect the potential groundwater recharge zones of Uckermark district and observe the groundwater level condition over a period of 21 years (2000–2020). Analytic Hierarchy Process has been used to locate the potential groundwater recharge zones and later a Long Short-Term Memory (LSTM) based model has been developed to forecast the seasonal groundwater level for the upcoming five years in the potential groundwater recharge zones based on observation data from groundwater measurement points. This enabled us to see the groundwater condition of Uckermark in near future and point out the necessary steps to be taken.

## Introduction

Groundwater is world’s biggest freshwater resource and crucial for agriculture and anthropogenic usage^1^. It is regarded as a vital freshwater source and during extended drought periods it becomes an aid^2^. When there is a lack in groundwater recharge, or a shortage in groundwater storage over a particular time in a specific area, the phenomenon can be termed as groundwater drought^3^. Globally, groundwater decrease has been noticed in many agriculturally significant regions^4^. In arid and semi-arid areas, groundwater is the means of survival for stream flow; thus, changes in groundwater systems affect watershed condition^5^. Massive drought occurrences in Europe from the twenty-first century had severe socio-economic consequences^6^. Since 1980, the frequency of drought has increased significantly in Germany and it will keep rising according to European Commission. This increasing drought frequency in Germany is ultimately causing increased evapotranspiration^7^. For instance, Brandenburg, one of the largest states of Germany, is characterized by its lowest rate of precipitation. The average annual temperature of Brandenburg is on an increase, and on the other hand, the mean average precipitation of the last four decades has remained almost the same^8^. Uckermark, a district of northeastern Brandenburg state of Germany is characterized by agriculture, as 63% of its total landmass (192,000 ha) is being used for this purpose. The region has one of the lowest precipitation rates in Germany (490–640 mm/year), indicating temperate continental climate^9^. Numerous groundwater observation gauges of the region indicate depletion in the groundwater table level over years of observation. The groundwater table fluctuation reasons are related to parameters such as groundwater level, soil type, temperature, and amount of precipitation^10^. One of the significant factors causing groundwater table depletion may be that the extraction rate is more than the recharge rate, which is also termed as groundwater stress^11,12^. Moreover, northeastern Brandenburg has been characterized by less than average annual precipitation, high air temperature during the growing period causing high evapotranspiration and low recharge rate of groundwater^13^. As an outcome, groundwater recharge regions are affected by the decaying availability of recharge water and decreasing groundwater level over time^14^. To understand the groundwater resources of a region, it is essential to get an understanding of essential hydrological and hydrogeological factors such as drainage density, geomorphology, landform characters, land use and land cover (LULC), slope, precipitation, soil types, and climatic conditions^15^. Remote Sensing (RS) provides an essential baseline on geophysical and hydrogeological components^16^. And techniques of geographic information system (GIS) such as weighted overlay analysis alongside structured decision-making technique like analytic hierarchy process (AHP) is popularly used to determine the potential zones for groundwater recharge^15,17–19^. On the other hand, groundwater level forecasting enables us to anticipate the water quality of non-sampled depth zones and evaluate the sustainability of remaining groundwater^20,21^. For the last couple of decades, machine learning methods have become popular in various RS related studies^22,23^. The significant characteristic of artificial neural networks (ANN) is that, they are usable for solving incomplete information problems without the analytical relationship between the input and output data. This feature has made ANN vital for complex relationship modeling between a huge range of variables^24^. Recurrent Neural Network (RNN) is a type of ANN that can process sequence data by elements and after that preserves a state for representation of the information at time steps. Now, Long Short-Term Memory (LSTM) is a popular form of RNN that can retain context over long spans and it has shown potential in hydrological time series prediction^25^. So, recurrent neural network (RNN) like long short-term memory (LSTM) can be used to predict the near-future condition of ground^26^. In this research initiative, an attempt has been made to determine the potential groundwater recharge zones of Uckermark district and forecast the district’s groundwater tables of the potential groundwater recharge zones during summer and winter. This research aims to predict the near-future scenario of groundwater levels and shows how the groundwater recharge scenario is changing in this heavily agro-based district of Germany.

## Study area

**Figure 1:**
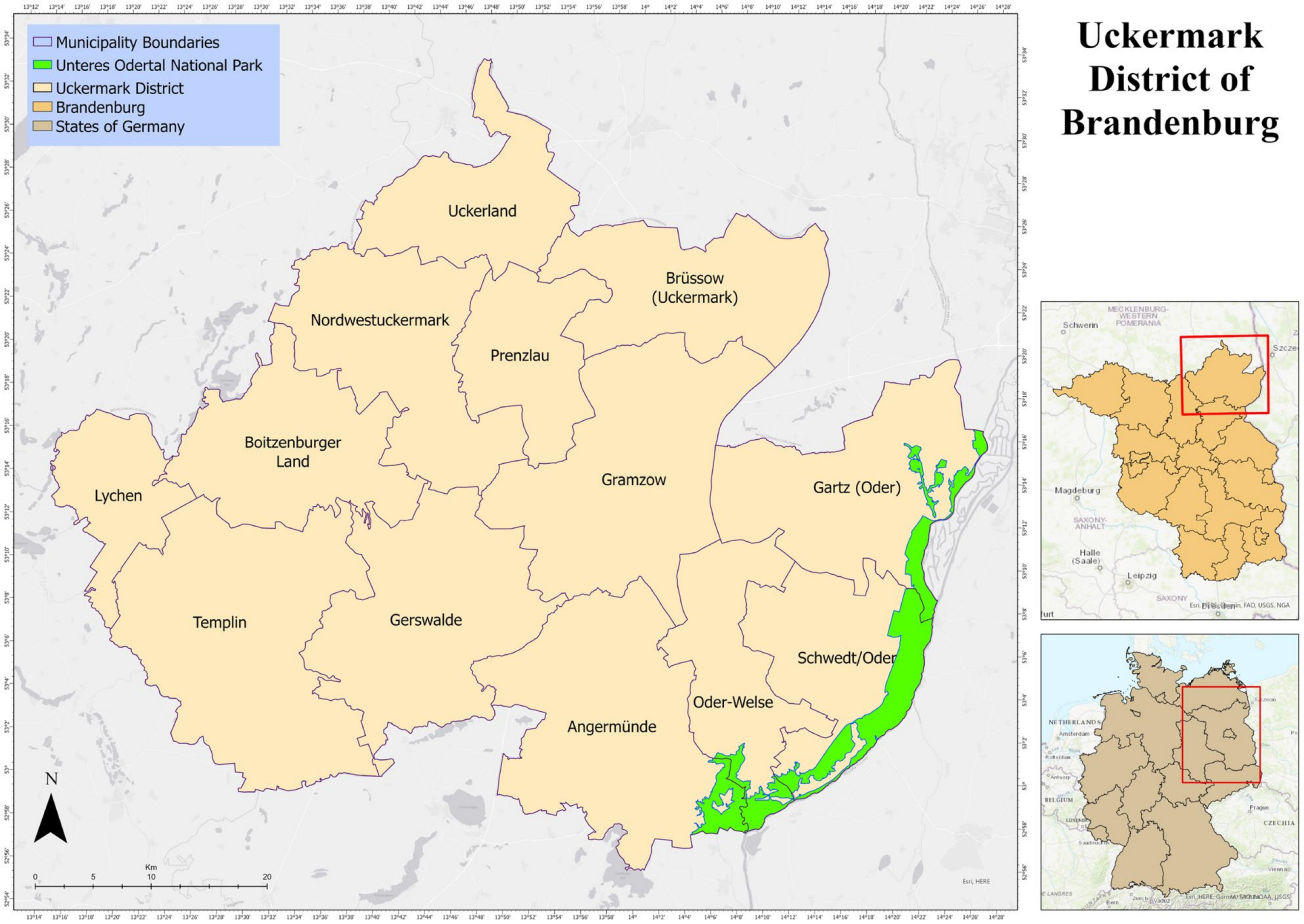
Uckermark District of Brandenburg, Germany.

Uckermark district, shown in Fig. 1, is the largest district of Brandenburg with about 3077 km$^2$ of area and a population of 118,689 (as of March 31, 2020). The name of the district is after the Ucker River which is a tributary of river Oder. The district shares the eastern border with Poland and the Unteres Odertal National Park. The capital of Germany, Berlin is situated about 100 kilometers south of the district. On the north, the district shares boundary with Mecklenburg–Vorpommern state of Germany as Uckermark is situated in the north-eastern edge of Brandenburg. The district is hydrologically significant being contained with about 600 lakes and 2800 km of rivers.

The positional benefits of Uckermark precisely lie in an extensive range of sectors. Major industries like mineral oil processing, paper production, mechanical engineering, metal processing, food sector, and wood processing plants are just as at home in Uckermark as innovative SMEs involved in regional handicrafts and trade. Agriculture is of high economic significance in the Uckermark district as it employs 7.5% of the district’s total labor force. The total agricultural area of the district is about 192,000 ha which is 63% of the total area of Uckermark and it makes Uckermark the most significant agrarian community of Brandenburg. The district is actively focused on organic farming. Therefore 55 enterprises work in 16,226 ha making it the largest connected ecological farming area of Europe. The region consists of Pleistocene glacial sediments which came from three large glaciations of Weichselian, Saalian and Elsterian. Uckermark’s regional groundwater structure is aligned with the Pleistocene glacial landscape, with different regional groundwater systems being delineated on the basis of large geomorphological structures such as glacial valleys, till upland regions and end moraines. Sediment types deposited over the study area consist of a range of clastic sediments of glacial fluvial origin and till^27,28^.

**Data used:** For the AHP, 8 different layers were generated using data from different sources. All sources for potential groundwater recharge zone detection have been explained in Table 2. For forecasting of groundwater level through LSTM, groundwater level data of 20 years (2000–2020) was provided by the State office for the Environment (LfU) Brandenburg.

## Methodology

### Workflow

Data was gathered to find potential groundwater recharge zone detection through analytic hierarchy process (AHP) using ArcGIS Pro 2.5. Eight geospatial data layers essential to detect potential groundwater recharge zones were gathered from various sources and used in this AHP based weighted overlay analysis for pointing out the zones suitable for groundwater recharge.

Then different potential groundwater recharge zones of the Uckermark district were separated to particularly focus on the groundwater levels of the specific areas. Groundwater measurement points from ’State Office for the Environment’ (Landesamt für Umwelt) of Brandenburg were separated.

Groundwater station data of 21 years (2000–2020) was provided by the State Office for the Environment (LfU) of Brandenburg state. Seasonal groundwater station data of summer (June–August) and winter (December–February) of each seasonal cycle of the provided 21 years were separated.

**Figure 2:**
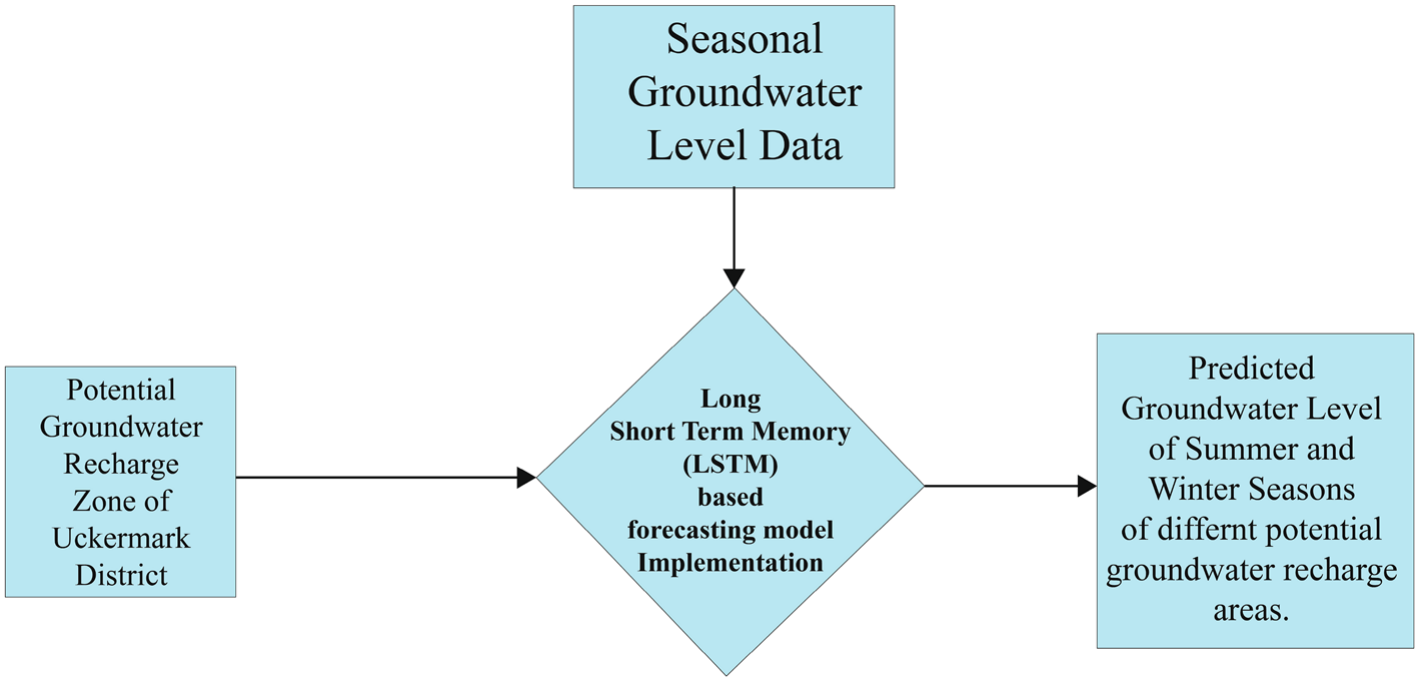
Workflow for seasonal groundwater level prediction.

A Long Short-Term Memory (LSTM) based forecasting model has been developed for seasonal groundwater level forecasting from 2021 to 2025 of the potential groundwater recharge zones belonging to the drought-prone area of the Uckermark district, described in Fig. 2.

### Analytic hierarchy process

Multi-criteria decision-making tools are used in GIS for various purposes. Analytic hierarchy process is a multiple criterion decision-making tool. It is an Eigenvalue approach to the pairwise comparison. It also provides a methodology to adjust the numeric scale for the measurement of quantitative as well as qualitative performances. The scale ranges from 1/9 for ’least valued than’, to 1 for ‘equal’, and to 9 for ‘most important’ covering the entire spectrum of the comparison^29^.

AHP is a multiple-criteria method based on the need for complex problems splitting into a hierarchical configuration of specific objective (goal), conditions (sub-criteria), and substitutes. AHP method application can be described in four simple steps^30^:

1. It is creating a hierarchical problem model for which a decision should be made. The goal is located at the top of the hierarchy, criteria and sub-criteria are put at the lower levels.
2. At each level of the hierarchy, contrast in pairs of elements is done, where the preferences of the decision-maker are expressed using Satty scale of relative importance levels^31^. The scale contains 5 levels and 4 sublevels, which verbally describe the strength, with equivalent numeric values on the scale of 1 to 9 (Table 1):

**Table 1:** Importance scale of analytic hierarchy process (AHP)^29,30^.

Importance	Definition	Explanation
1	Equally importance	Both elements have equal contribution to the objective
3	Moderately importance	Modest advantage of the one element compared to the other
5	Strong importance	Strong favoring of one component contrasted to the other
7	Very strong importance	One element is strongly favored and has supremacy in practice, compared to the other component
9	Extreme importance	One component is favored in comparison with the other, based on strongly proved evidence and facts
2, 4, 6, 8	Inter-values	The values that are in between the major importance levels
3. The analysis of comparative importance to the elements from each hierarchic level is applied for measurement of local criteria, sub-criteria and alternatives. Later total priorities of the substitutes are synthesized.
4. Finally sensitivity analysis is executed.

### Potential groundwater research zone detection

**Figure 3:**
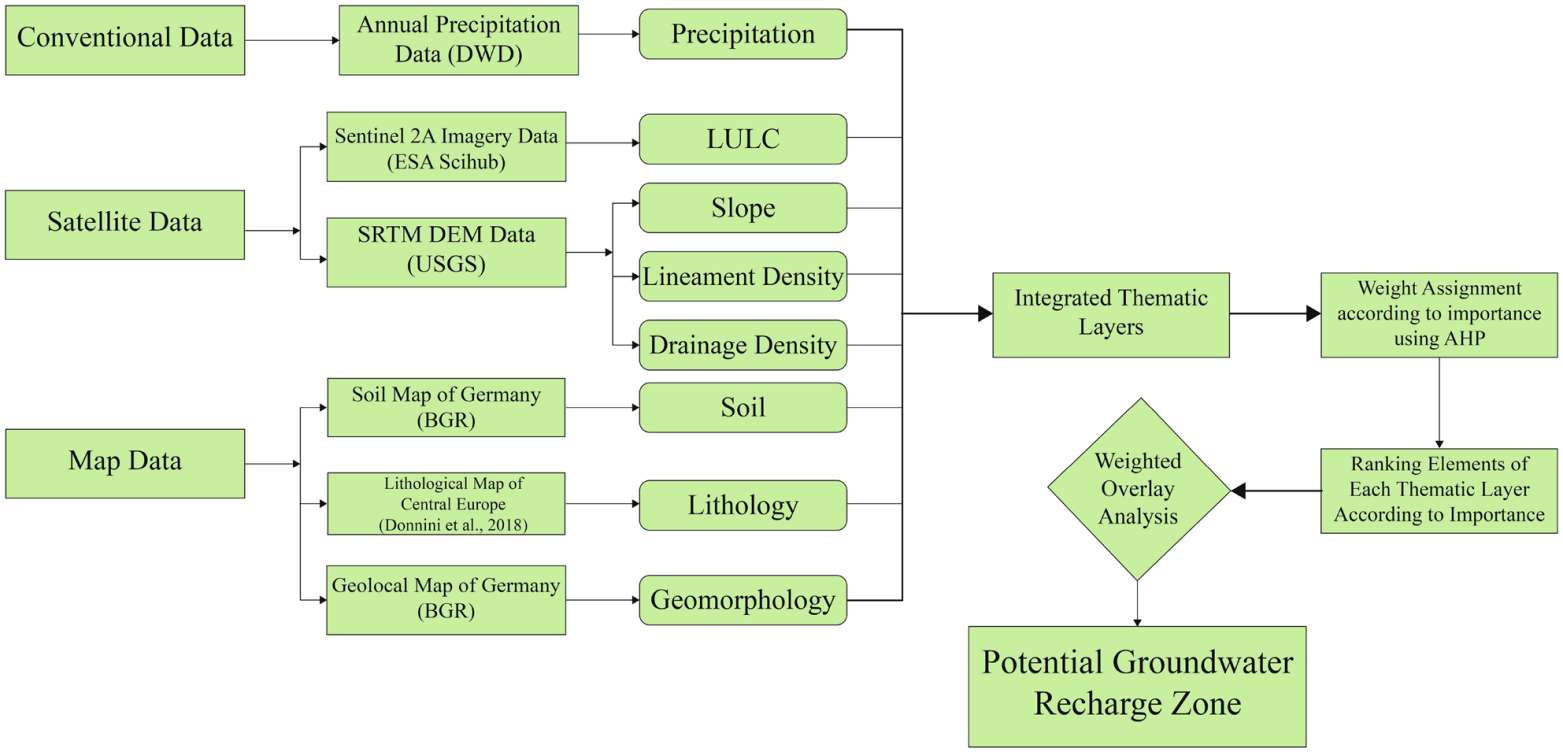
Workflow for potential groundwater recharge zone detection.

For potential groundwater recharge zone detection, first necessary data for thematic layer generation was collected. After going through important literature^15,17,18,32^ regarding potential groundwater zone delineation, six datasets were selected for analysis. Among the six datasets one was conventional data (annual precipitation data), two were satellite imagery data (ESA’s multispectral data of Sentinel 2 satellite and NASA’s SRTM DEM) and three were pre-processed map data (soil, lithology, geology). From the precipitation data, the precipitation layer is generated. Land Use and Land Cover layer of Uckermark (2020) is generated from the Sentinel 2 satellite imagery. 3 layers, slope, lineament, and drainage density are generated from SRTM DEM data. Soil type layer is generated from Soil Map layer of Germany. The lithology layer is extracted from the lithological map of Central Europe produced in 2020^33^. The geomorphological layer is extracted from the geological map of Germany. So, a total of eight thematic layers have been produced from six datasets. The weight of the layers was assigned according to their internal relationship, which sets up each layer’s hierarchical rank. Later all the elements of the layers were ranked according to their importance regarding groundwater recharge potential. And finally, potential groundwater recharge zones have been detected using the weighted overlay analysis tool of ArcGIS pro where the layers were put according to their hierarchic rank. Figure 3 provides a clear view of the workflow.

### Data sources for potential groundwater recharge zone detection

Literature studies have been conducted to determine the inter-relationship between the geospatial layers of influencing factors for potential groundwater recharge zone detection^15,34–37^. Table 2 is showing all used layers of influencing factors and their sources.

**Table 2:** Data sources for AHP.

Layer name	Source	Resolution
Land Use Land Cover (LULC)	Sentinel-2A, European Space Agency (https://scihub.copernicus.eu/)	10 m
Precipitation	German Meteorological Service (cdc.dwd.de)	Gross annual
Slope	SRTM-DEM, USGS (https://earthexplorer.usgs.gov/)	30 m
Lineament density	SRTM-DEM, USGS (https://earthexplorer.usgs.gov/)	30 m
Lithology	Geo-LiM: A New Geo-Lithological Map for Central Europe (https://zenodo.org/record/3530257)	1:2,000,000
Geomorphology	Federal Institute of Geosciences and Natural Resources (www.bgr.bund.de)	1:1,000,000
Soil types	Federal Institute of Geosciences and Natural Resources (www.bgr.bund.de)	1:200,000
Drainage density	SRTM-DEM, USGS (https://earthexplorer.usgs.gov/)	30 m

### Layer prioritization for potential groundwater recharge zone detection

**Figure 4:**
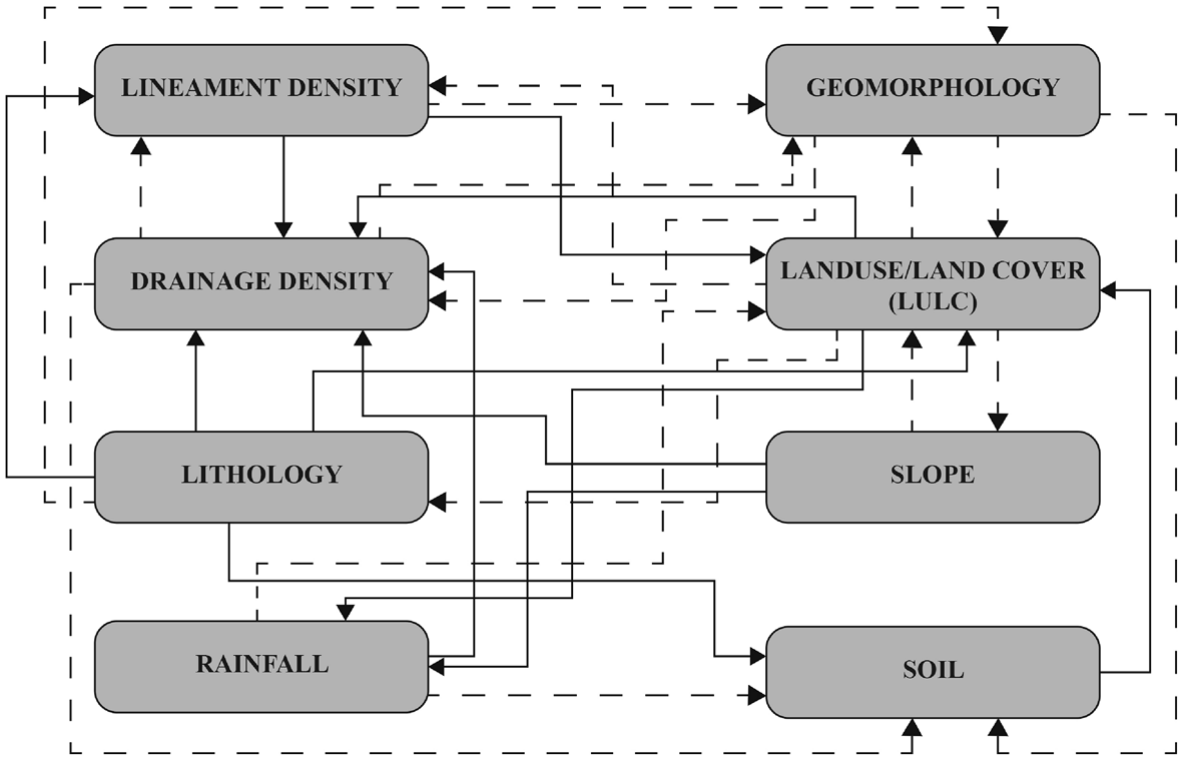
Inter-relationship between the influencing factors of the potential groundwater recharge zone.

After extensive literature studies^11,15,18,34^, the inter-relationships between the parameters have been determined and their inter-relationship was divided into two categories—‘Major Effects’ and ‘Minor Effects’. The relationship between the elements is shown in Fig. 4. The factors in Major Effect (A) were assigned a value of 1 and Minor Effect (B) was assigned a value of 0.5. The gross score (A + B) of both major (A) and minor (B) were considered for estimation of the relative values. Hence, the rank of each factor was scored using the following equation^15^:

1 $$\begin{aligned} \text{ Score } =\left\{ \frac{(A+B)}{\sum (A+B)}\right\} \times 100. \end{aligned}$$

1 and 0.5 were assigned as scores to the variables depending on their impact on the recharge of groundwater^15,38^. The score of each factor was reclassified to sub-factors (Table 3). The sub-factor was used to measure the weight of each influencing factor.

For instance, in the study area, lithology (pit, silt, sand) has major impact on four influencing factors of groundwater recharge potential (lineament, land use, soil and drainage) and minor impact on geomorphology of the area. Thus, 4.5 was assigned to lithology. Similarly, slope has a large impact on rainfall and drainage density and small influence on LULC; therefore, combining the major and minor impact, a total weight of 2.5 is assigned to slope. The inter-relationship between these factors changes with context and type of features available to use^39^.

**Table 3:** Ranking of influencing factors.

Factor	Major effects (A)	Minor effects (B)	Proposed relative rates (A + B)	Proposed score of each influencing factor
Lineament density	1 (D)+ 1 (LULC)	0.5 (Geo)	2.5	12
LULC	1 (D)+1 (P)	0.5 (Sl) + 0.5(Geo) + 0.5(Lin) + 0.5(Lith)	4	20
Lithology	1 (D) +1 (Lin)+ 1 (LULC) + 1 (So)	0.5 (Geo)	4.5	22
Drainage density	1 (LULC)	0.5 (Geo)+ 0.5(So) + 0.5(Lin)	2.5	12
Slope	1 (P)+1 (D)	0.5(LULC)	2.5	12
Precipitation	1 (D)	0.5(LULC) + 0.5(So)	2	10
Soil	1 (LULC)		1	5
Geomorphology		0.5 (LULC) + 0.5(So) + 0.5(D)	1.5	7
			$\sum$ = 20.5	$\sum$ = 100

In the next step, weight of every influencing factor was calculated using equal division method and ranks were assigned to each sub layer of all factors. Table 4 contains weight and rank of sub layers of all influencing factors. Every thematic layer was assigned with weights visualizing different polygon of individual characteristics.

### Long short-term memory (LSTM)

Long short-term memory network (LSTM) is a special kind of RNN, which is capable of learning long-term dependencies. LSTM was first introduced in 1997^40^ and was refined and popularized by many researchers^26,41^. LSTM works well on a large variety of problems and is now popularly used in water level prediction and forecasting, stock market forecasting etc^26,42^. LSTMs are particularly designed to avoid long-term dependency problems. Recollecting information for longer periods is its default behavior. The first step in LSTM is to decide what information to discard from the cell. This decision is taken by a sigmoid layer called the “forget gate layer.” It takes in h_t-1_ and x_1_ as inputs and outputs a number between 0 and 1 for each number in the cell state C_t-1_. Where 1 represents keeping the information and 0 illustrates discarding the data^40^.

2 $$\begin{aligned} f_{t}=\sigma \left( W_{f} \cdot \left[ h_{t-1}, x_{t}\right] +b_{f}\right). \end{aligned}$$

The next step is to decide what information is going to be stored in the cell. This has two parts—first, a sigmoid layer called the “input gate layer” decides which values to update. Then a tanh layer generates a vector of new candidate values, C_t_ , that could be added to the state^40^.

3 $$\begin{aligned} i_{t}=\sigma \left( W_{i} \cdot \left[ h_{t-1}, x_{t}\right] +b_{i}\right), \end{aligned}$$

4 $$\begin{aligned} \tilde{\mathrm {C}}_{t}=\tanh \left( W_{c} \cdot \left[ h_{t-1}, x_{t}\right] +b_{c}\right) \end{aligned}$$

**Table 4:** Prioritizing sub layers for AHP.

Major layers	Sub layers	Ranking
Lithology	Peat	9
	Gravel	7
	Diamicton	7
	Silt	7
	Sand	7
	Sedimentary rocks	5
	Clay	5
LULC	Water bodies	9
	Agriculture	7
	Forest area	7
	Grass land	7
	Barren lands	3
	Urban areas/settlements	1
Drainage density	< 74.36 km$^{-1}$	9
	74.36–148.7 km$^{-1}$	8
	148.7–222.07 km$^{-1}$	5
	222.07–297.43 km$^{-1}$	3
	> 297.43 km$^{-1}$	1
Lineament density	> 0.295 km$^{-1}$	9
	0.222–0.295 km$^{-1}$	7
	0.147–0.222 km$^{-1}$	5
	0.07–0.147 km$^{-1}$	3
	< 0.07 km$^{-1}$	1
Slope	0°–1°	9
	1°–2°	7
	2°–3°	5
	3°–5°	3
	> 5°	1
Precipitation	> 538.17 mm	9
	486.35–538.17 mm	7
	434.54–486.35 mm	5
	382.72–434.54 mm	3
	< 382.72 mm	1
Geomorphology	Lime stones	9
	Fluvial plain	7
	Glacial plain	7
	Telmatic Fen	5
	Rock formations	3
	Marine debris	1
Soil	Alluvial soil	9
	Fen soil	7
	Parabrown earth	7
	Podzol	5
	Ribbon parabrown earth	5
	Pale earth	3
	Podzol brown earth	3
	Brown earth	3

Then old cell state Ct-1 is upgraded to the new cell state Ct. The old state is multiplied by ft, forgetting the information decided to forget earlier. Then i_t_*C_t_ is added. These are the new candidate values, scaled by how much it is determined to update each state value^40^

5 $$\begin{aligned} C_{t}=f_{t} * C_{t-1}+i_{t} * \tilde{\mathrm {C}}_{t}. \end{aligned}$$

Finally, the output is needed to be decided. This output will be based on the cell state, but it will be a filtered version. First, a sigmoid layer is run, which decides what parts of the cell state will output. Then, the cell state is put through, and then the sigmoid gate output is multiplied, so that only decided parts become the outputs^40^.

6 $$\begin{aligned} o_{t}=\sigma \left( W_{0}\left[ h_{t-1}, x_{t}\right] +b_{0}\right. \end{aligned}$$

7 $$\begin{aligned} h_{t}=o_{t} \times \tanh \left( C_{t}\right) . \end{aligned}$$

### Groundwater data collection for LSTM

**Figure 5:**
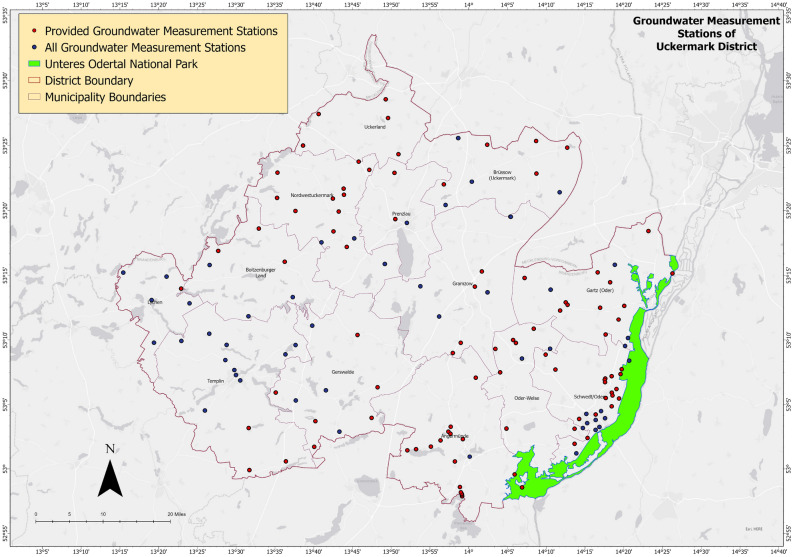
Groundwater measurement stations of Uckermark district.

Groundwater table data of Uckermark has been provided by the State office for the Environment (LfU) Brandenburg. LfU has provided continuous groundwater table data of 21 years (2000–2020). Among 180 stations, groundwater table depth data of 100 stations were provided by LfU. Data from 80 stations were not provided because most of the 80 stations have short-term observations or are situated in an urban area. Among the 100 stations, 5 were discarded because of a shortage in data. The forecasting was done using groundwater table data of 95 groundwater measurement stations scattered around Uckermark (Fig.  5).

### LSTM prediction model setup

To set up LSTM based prediction model, the following steps are followed:

1. Firstly, the summer and winter datasets were imported separately and converted to a data frame using pandas.
2. Specific data columns were selected from both data frames for sequencing datasets using NumPy.
3. Then the sequence dataset was put into the ‘traintestsplit’ module of sklearn. The test size was 70% and the training size was 30%.
4. After that, the LSTM model was imported from PyTorch. In this model after many trials and errors, the following criteria were set:
  - Input dimension: 1
  - Hidden dimension: 200
  - Epochs: 200
  - Learning rate: 0.001

### Model testing and validation

The performance of the LSTM model has been tasted using the standard performance index from Nash and Stucliffe, popularly known as Nash–Sutcliffe Efficiency^43^. The simulation used for the model’s efficiency is conducted using the groundwater level dataset of 95 groundwater stations of Uckermark district of the summer season between (2000–2014). Datasets of summer is used because the groundwater level is high during summer regarding to other seasons in Uckermark. And before forecasting the upcoming years it is necessary to validate the model with existing datasets and proper validation methods. The Nash-Stucliffe Efficiency coefficient (NSE):

8 $$\begin{aligned} NSE= 1-\frac{\sum _{j=1}^{n}{(X-Y)}^2}{(X-Z)}, \end{aligned}$$

where, X is observed groundwater level (m), Z is the mean of the observed values and Y is the predicted values from the LSTM based groundwater forecasting model. The value found for NSE is 0.6768, which is considered good for simulation results^44,45^.

To evaluate the fitness of the model, besides the NSE the coefficient of determination (R$^2$), mean square error (MSE) and mean absolute error were used. The R$^2$ value indicates a good output if the value is greater than 0.542^42^. Low MSE and MAE indicates satisfactory fitting of the model^43^. The R$^2$ value for the existing LSTM based forecasting model is 0.87583709, MSE is 3.1357 and MAE is 1.8724. All these outputs indicates a well fitted model for forecasting groundwater level. To visualize how closely the prediction model has worked the summer dataset of from 2000 upto 2014 has been trained and a forecasting has been made for summer 2015 and the following outcome have been achieved:

**Figure 6:**
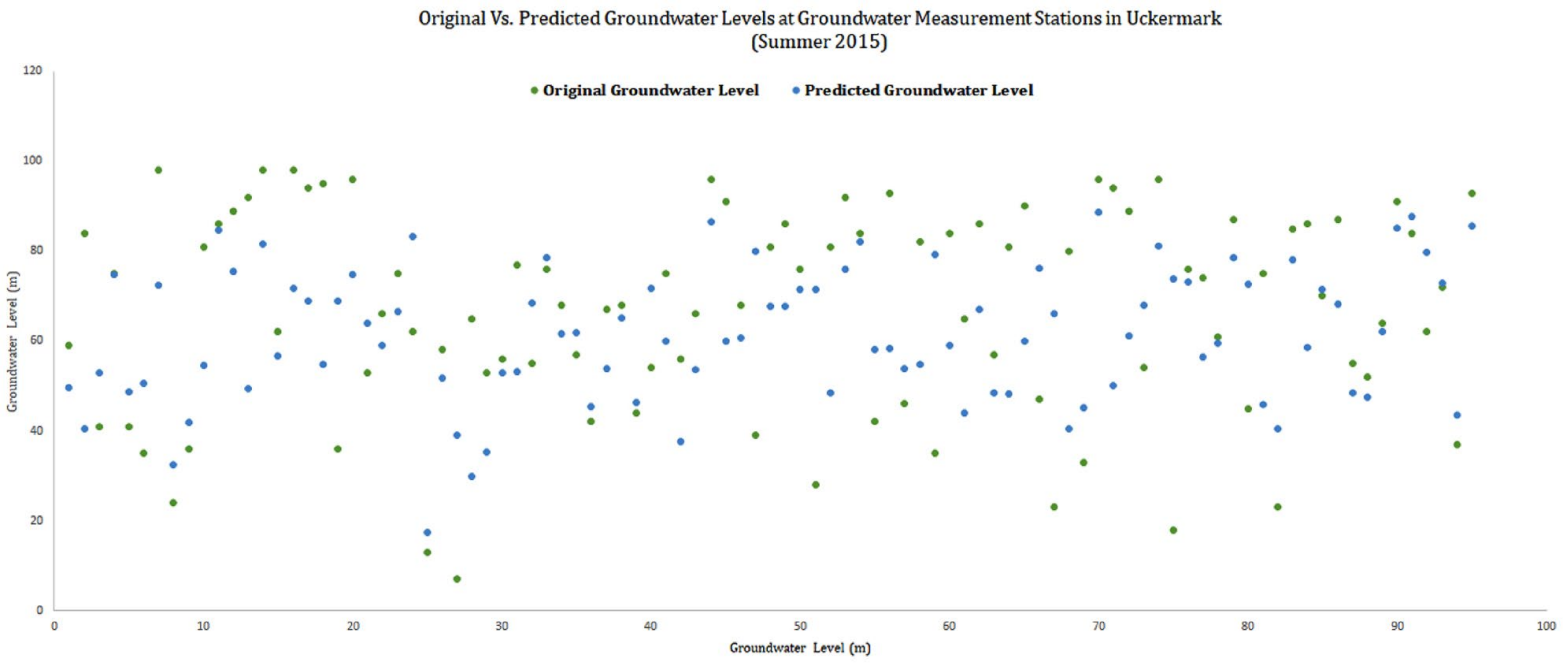
Original and predicted groundwater data of 95 stations.

The graph in Fig. 6 shows clarity about the precision of the prediction. The predictions gave a good assumption about the groundwater levels of Summer 2015 in Uckermark.

## Results

The generated 8 layers for potential groundwater zone detection are shown in four images.

**Figure 7:**
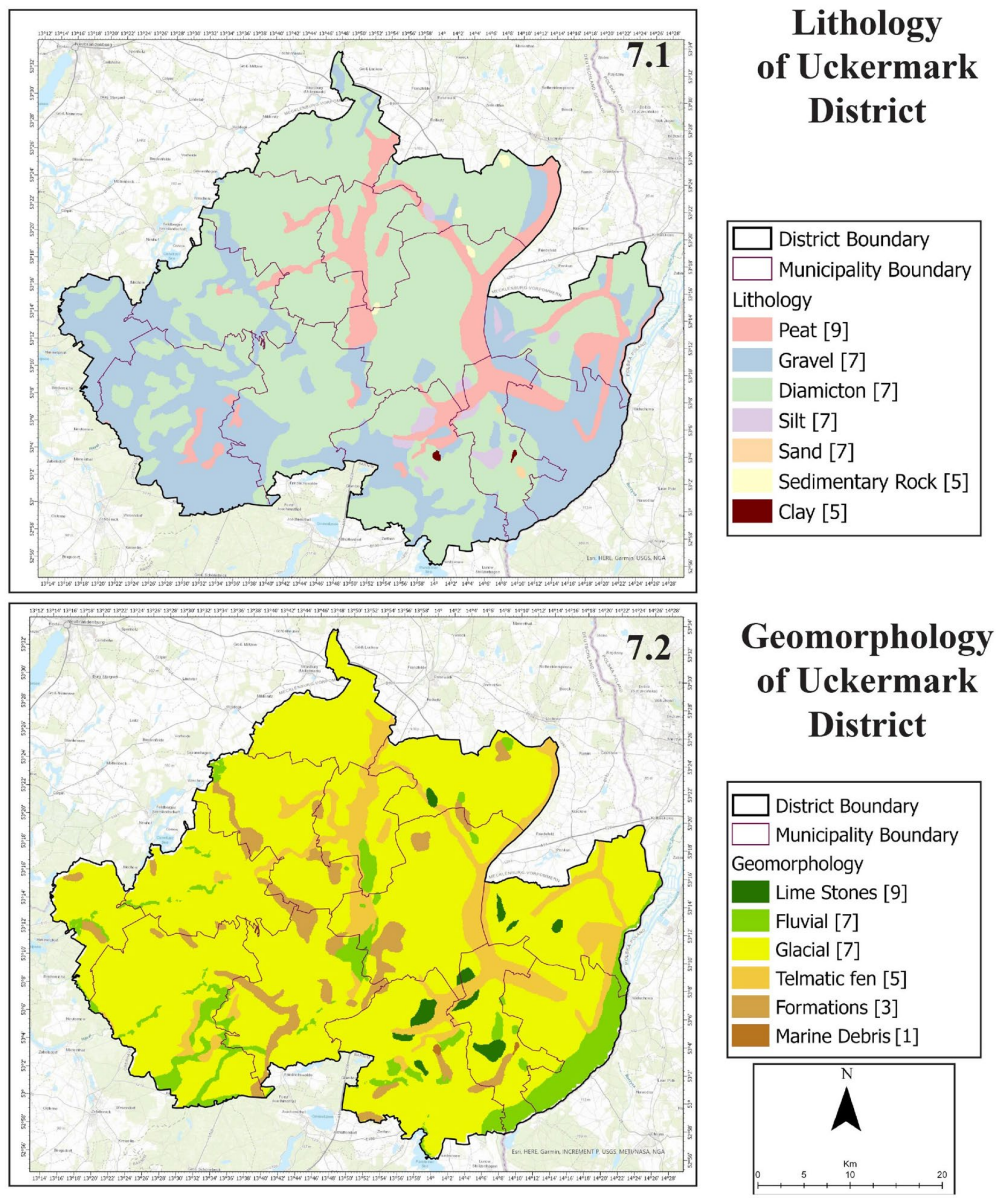
Lithology (7.1) and geomorphology (7.2) of Uckermark District.

From the lithology map of Uckermark (Fig. 7, 7.1), a diamicton type of lithological setting characterizes almost the maximum portion of Uckermark. The next most significant lithological formation gravel formations and followed by peat formations. A small part of land constitutes of Silt, Sand, Sedimentary Rock and Clay formations. Geomorphological (Fig. 7, 7.2) settings of Uckermark show that Glacial sedimentary environments characterize this district. The whole Unteres Odertal National Park area and some aquatic parts in the central region are of fluvial sedimentary environment.

**Figure 8:**
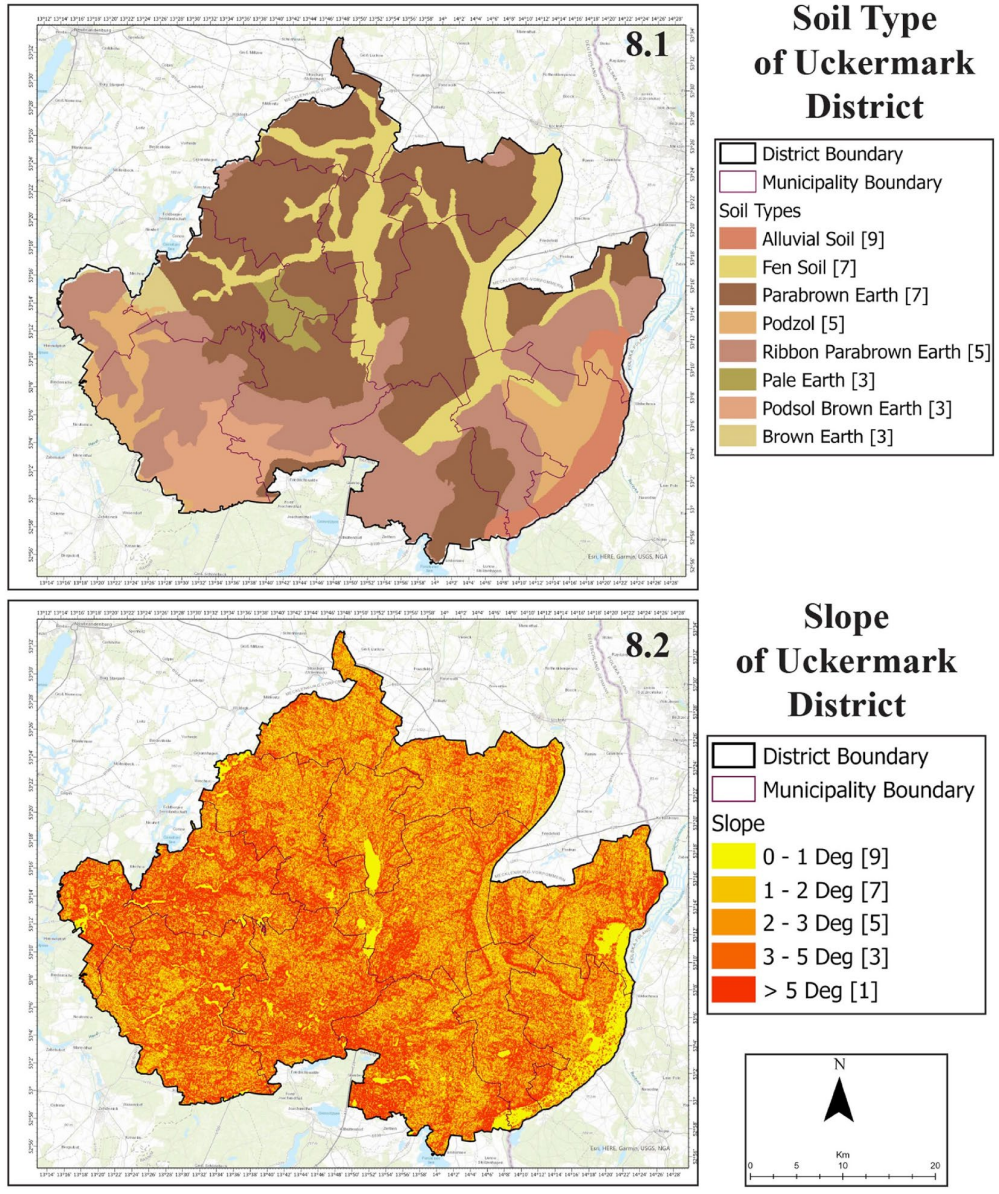
Soil type (8.1) and slope (8.2) of Uckermark District.

A significant portion of the land has telematic fen and other mixed geomorphological formations (Fig. 8, 8.1). The maximum soil of Uckermark is para brown earth. Followed by comes fen soils, podzol, pale earth, alluvial soil, etc. All these soil types are habitable for agriculture. The Unteres Odertal National Park in the east of Uckermark is situated in lowlands with 0°–1° of slope (Fig. 8, 8.2). However, most of the Uckermark has slopes between 1° and 5° and some areas are more than 5°.

**Figure 9:**
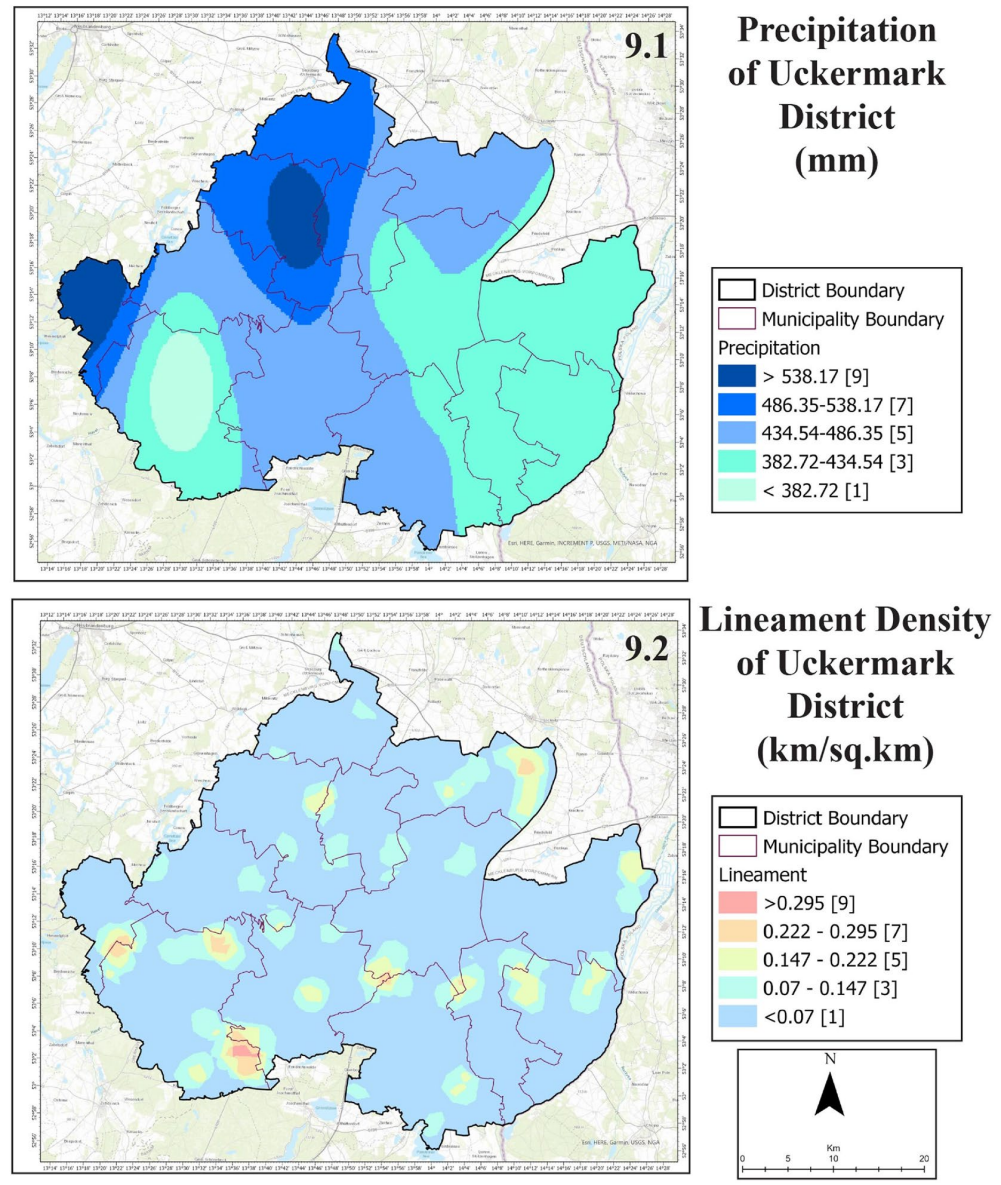
Precipitation (9.1) and lineament density (9.2) of Uckermark District.

Gross annual precipitation has a range of 382.72 mm to 538.17 mm in Uckermark (Fig. 9, 9.1). Despite being prone to drought, the northern portion of Uckermark had very high rainfall in 2020. The western part of Uckermark also had a high rate of precipitation in 2020. The eastern part and south-eastern part were low in precipitation rates. Uckermark district does not have high lineament density (Fig. 9, 9.2). Some discrete areas in southern Uckermark and northeastern Uckermark have slightly more lineament than other areas of the district.

**Figure 10:**
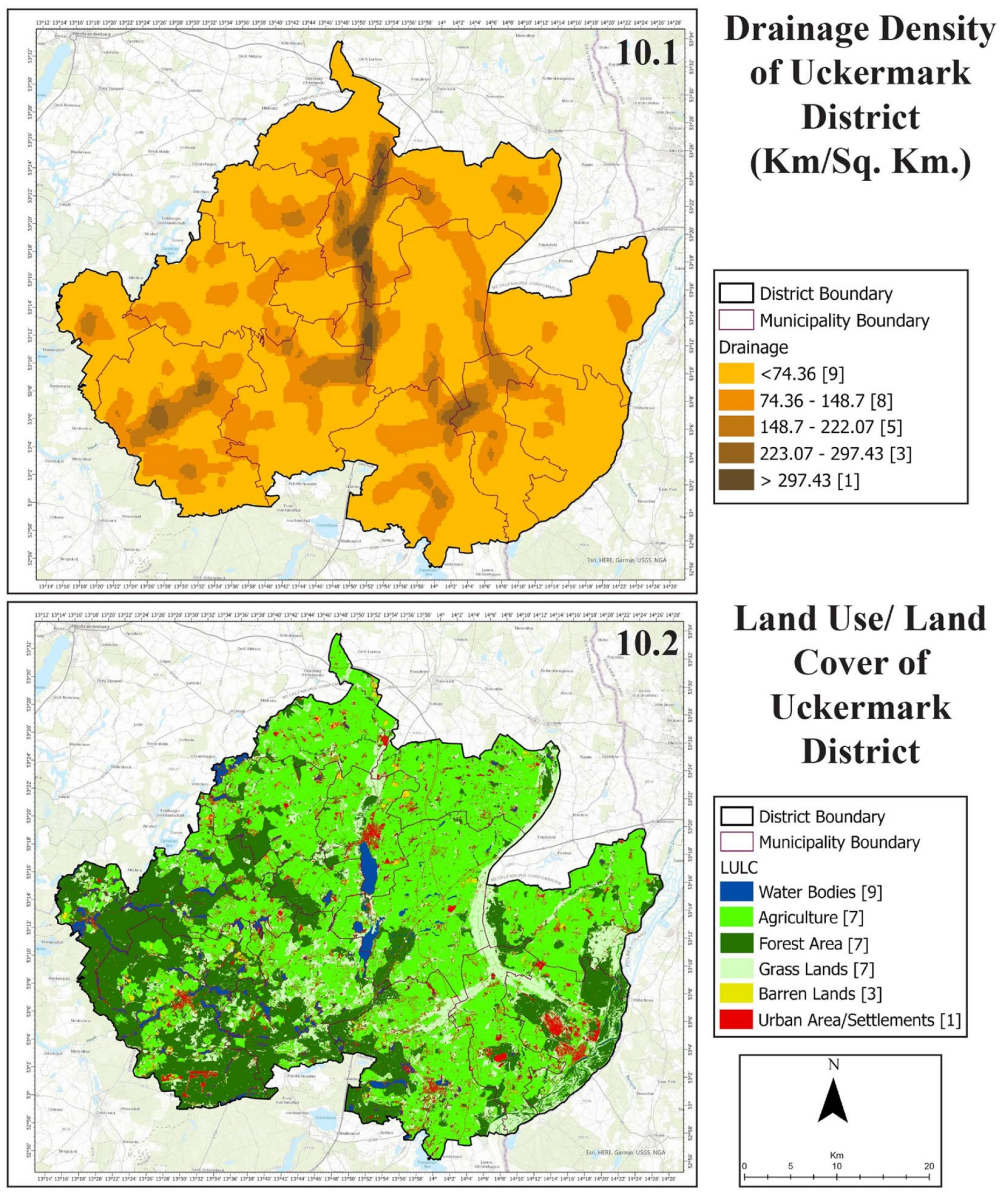
Drainage density (10.1) and LULC (10.2) of Uckermark District.

The central part of Uckermark is high in drainage density (Fig. 10, 10.1). Some major lakes of Uckermark are situated in this region- Unteruckersee, Sternhagener See, Potzlower See, and Oberuckersee. Also, some discrete areas of southeastern Uckermark are high in drainage density due to the presence of some major lakes- Fährsee, Templiner See, Lübbesee and Röddelinsee. Agriculture covers most of Uckermark with a total area of 1934 km$^2$ (Fig. 10, 10.2). Followed by forest area and grass lands which in total covers an area of 813 km$^2$. Urban settlements and barren grounds cover an area around 256 km$^2$. Water bodies cover the lowest amount of area with 74 km$^2$. It is clear from the analysis that greenery covers around 2747 km$^2$ of area, representing almost 89% of the total area of the Uckermark district. Hence water resources play an essential role in keeping this green region greener. After taking the layers mentioned above and their elements through the analytic hierarchy process, potential groundwater recharge zones of the Uckermark district are detected:

**Figure 11:**
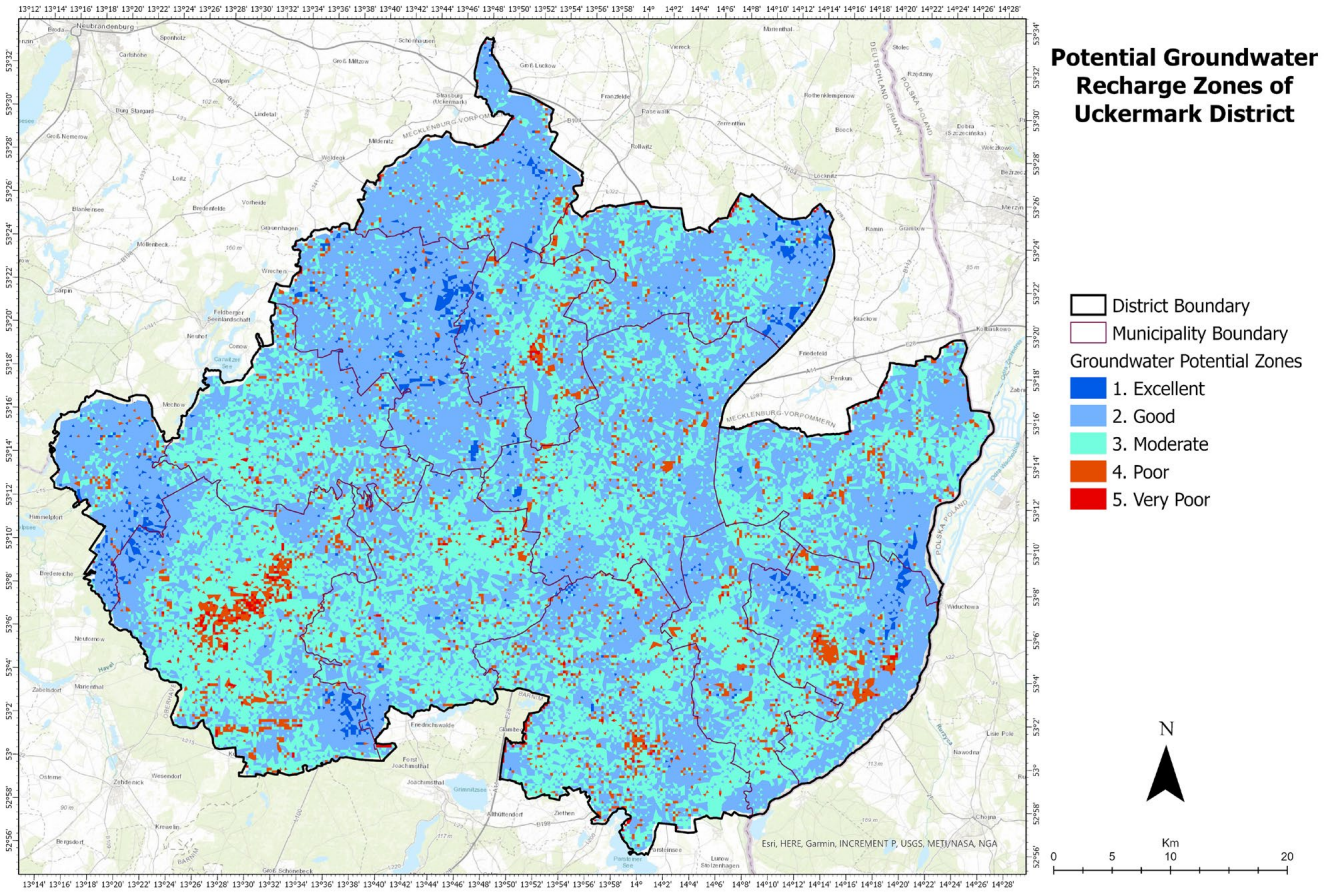
Potential groundwater recharge zones of Uckermark District.

From the analysis (Fig. 11), we can observe, about 53% of the total land of the Uckermark district is good for groundwater recharge. About 40% of Uckermark are moderately potential for groundwater recharge. About 4% of the total area possesses poor groundwater recharge potential and 1% of the total area is very poor for groundwater recharge. Sites with poor and very poor groundwater recharge potential are in urban areas. Excellent and good groundwater recharge potential sites are in agriculture and forest lands. Areas with moderate groundwater recharge potential are scattered around the catchments of lakes, agricultural lands, forest areas etc.

### Seasonal groundwater level prediction

**Figure 12:**
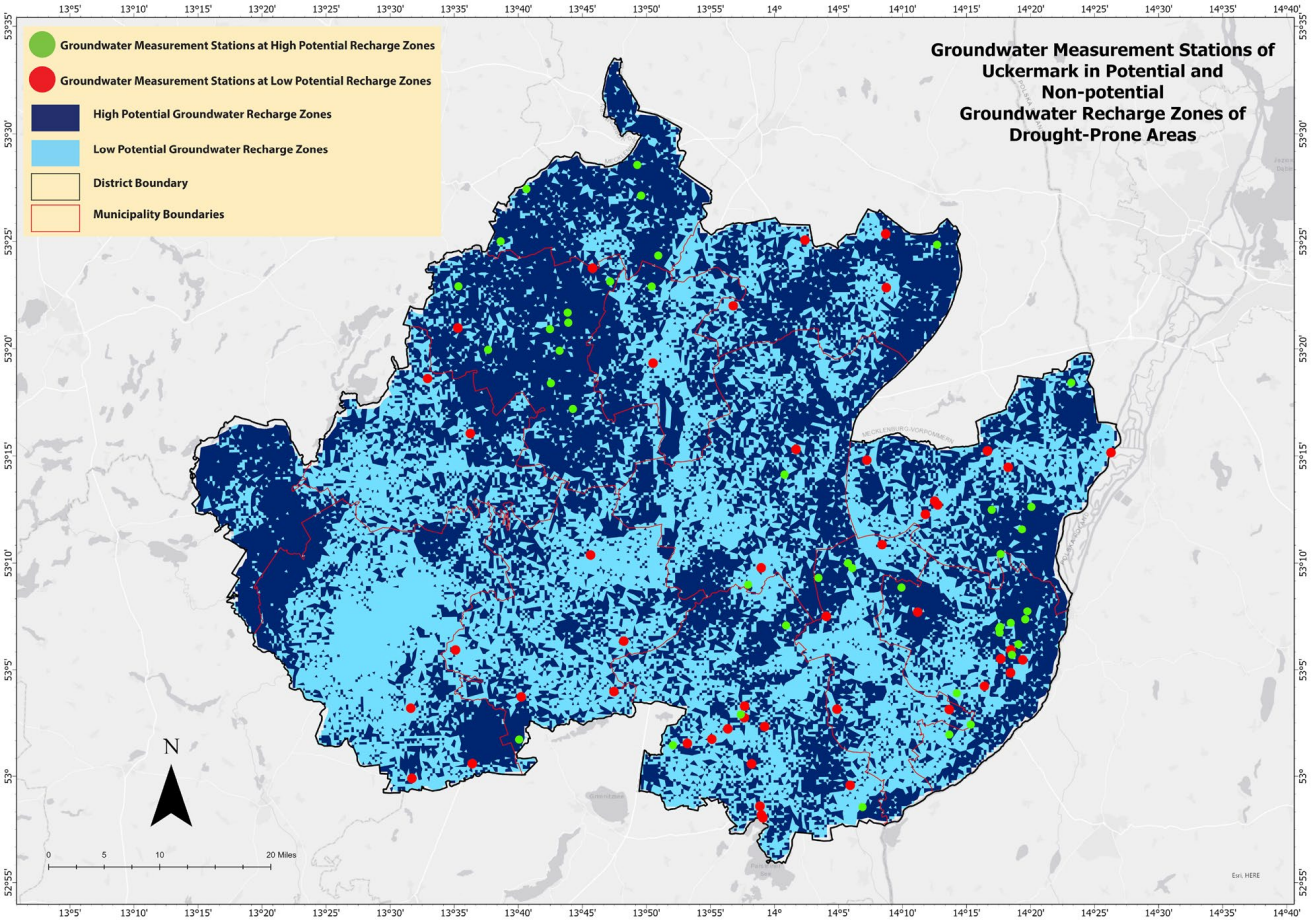
Potential and less potential groundwater recharge zones.

For this research, excellent and good groundwater potential areas (Fig. 12) has been considered as potential groundwater recharge areas. Moderate, poor, and very poor areas (Fig. 12) has been being considered as less potential groundwater recharge areas. If we separate the potential and non-potential groundwater recharge zones, we get 42 groundwater measurement stations in the potential groundwater recharge zone. And there are 47 groundwater measurement stations in the less potential groundwater recharge zone.

After visualizing summer and winter groundwater levels from interpolation of the station data, we get some insight about the groundwater level in the past, and what is going to be the scenario soon.

**Figure 13:**
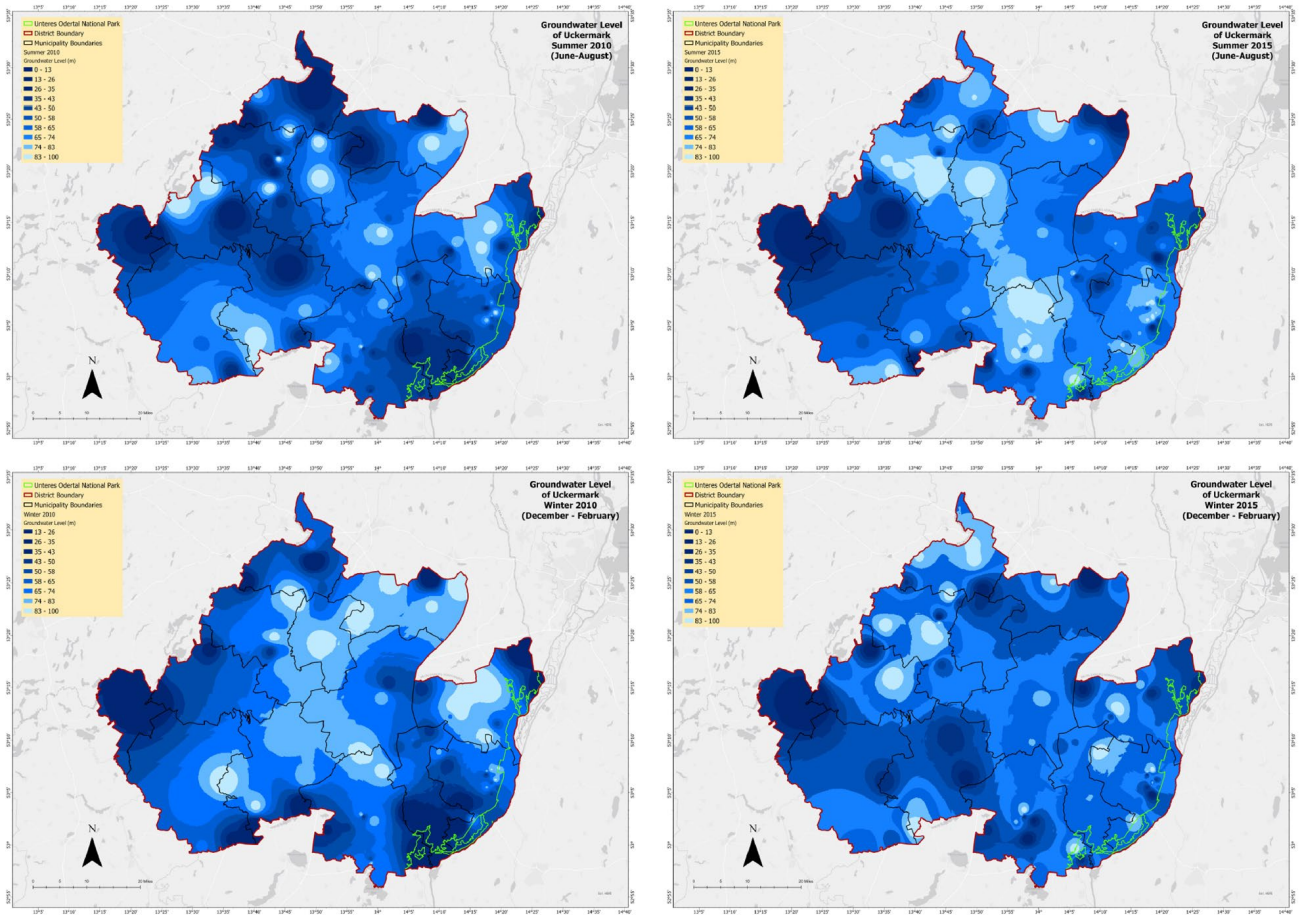
Groundwater level conditions of two seasons (2010, 2015) in Uckermark.

In 2010 (Fig. 13), we can observe a significant decrease in groundwater level, in winter. Starting from the central to north-eastern part there was a trend of groundwater decrease during winter. In summer, especially in the south-western and north-eastern part, there are some decreasing groundwater levels visible. In 2015 (Fig. 13), there was a decreasing trend of the groundwater level in Summer, mostly from the south-eastern part of Uckermark to the northern part. But during winter, the groundwater level was good except for some regions in the northern and north-eastern part of Uckermark.

**Figure 14:**
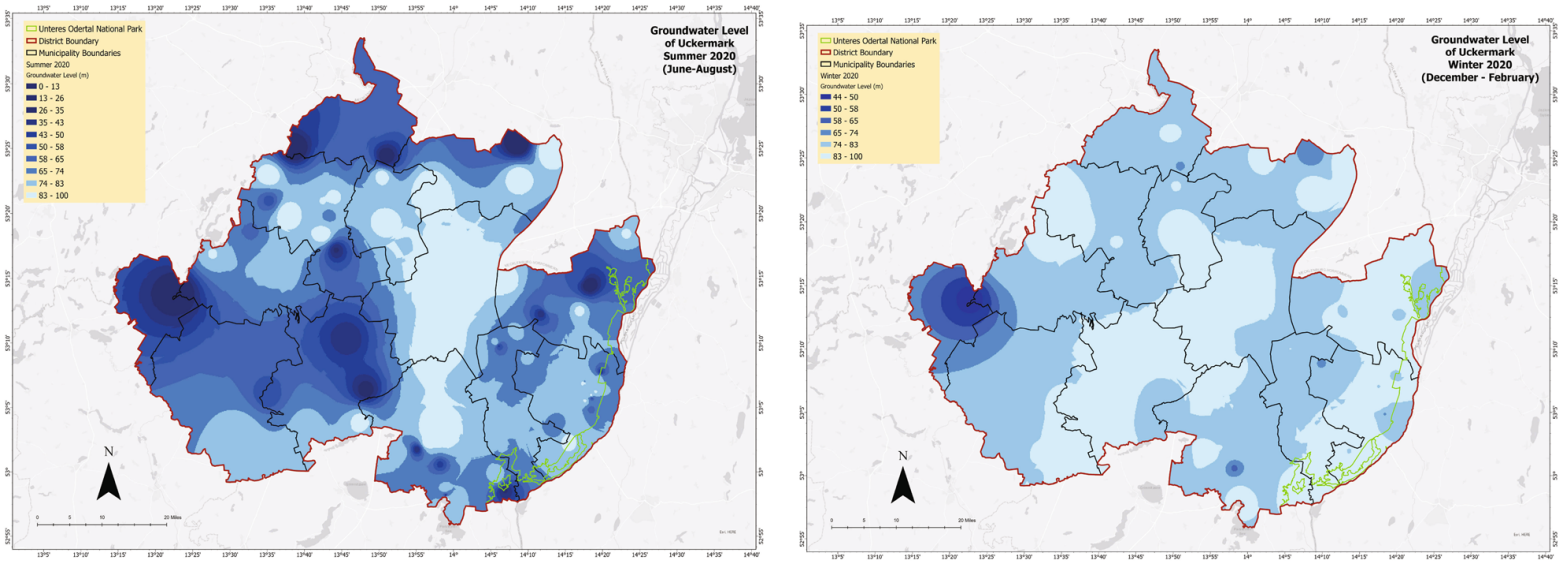
Groundwater level conditions of two seasons (2020) in Uckermark.

In 2020 (Fig. 14) there is a significant fall in the groundwater level in both summer and winter. During summer, the trend was prevailing from the southeast region to the northern Uckermark. But in winter the overall groundwater level of Uckermark shows a decrease and the groundwater level also shows a decay.

**Figure 15:**
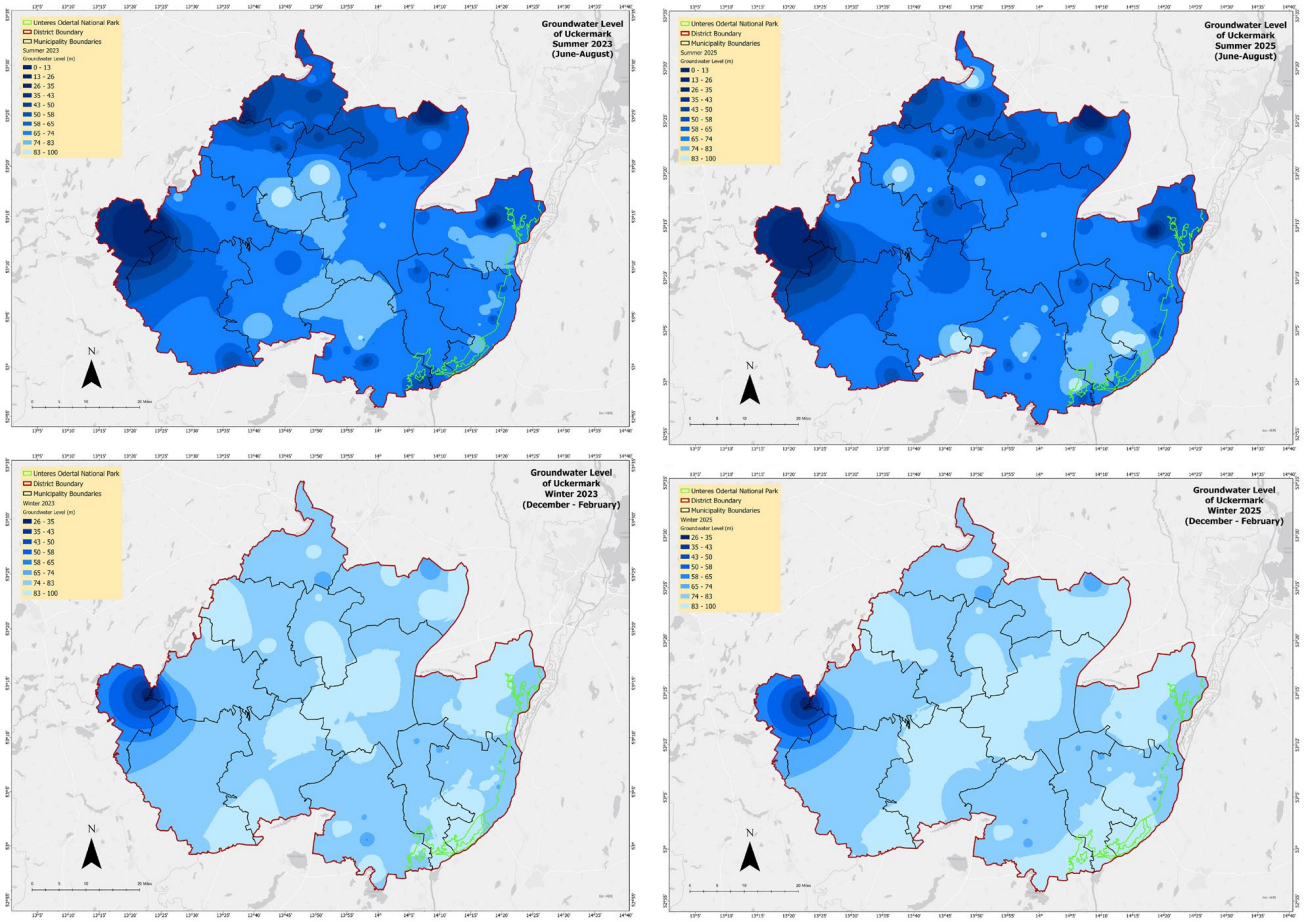
Predicted groundwater level conditions of two seasons (2023, 2025) in Uckermark.

The prediction model shows, in 2023 (Fig. 15) a decrease in groundwater level in winter around the whole of Uckermark except some parts in the western Uckermark which is not affected by drought. The northern, the middle and the south-eastern parts will adversely face winter groundwater drought. The groundwater level of Uckermark shows decreased groundwater level during winter in 2025 (Fig. 14). In summer, the south-eastern part is predicted to be drier. So, ultimately from the observations, we can say that the winter of Uckermark is drying rapidly and hence, the groundwater level is also decreasing.

## Conclusion

Uckermark is a district that has not faced any drastic change in land-use patterns over the last 200 years. Dense forests and agriculture have been the major land-use type of this region since the Prussian empire^46^.The observations of different geomorphological, lithological and land cover factors clearly state that, the soil and its elements existing in Uckermark can inhibit water for a good amount of time, but water takes time in this region to go through soil layers to the aquifers. On the other hand, the dry weather and insufficient rainfall are a reason for the water present on the surface to evaporate in the atmosphere. A study by Gutzler et al.^47^ suggests, the agriculture yield of Brandenburg is increasing with time and Uckermark is one of the major producing districts of the state. The irrigation water demand (IWD) of the state is more than 25%. This issue is critical regarding groundwater recharge and groundwater height. The study also shows, the pattern of rainfall in the region is also shifting from early to late winter despite having one of the lowest precipitations in Germany. This is resulting in an increase in groundwater extraction for irrigation in the region. If the balance of groundwater recharge and extraction rates are not maintained, there would not be enough water in near future for irrigation and major groundwater stress will occur due to heavy agriculture activities. This study suggests agriculture is not the only factor in Uckermark for decreasing groundwater level. Heavy oil refining, timber-based industries, and urban usage are also responsible for the decrease in groundwater level. Because, in the south-eastern part of Uckermark, there is decreasing trend of groundwater levels both in summer and winter, and the decrease is significant for both seasons. And, a better LULC classification method^48^.The south-eastern part of Uckermark is characterized by industrial activities, urban settlement, the presence of reserved national parks, and plenty of grasslands. This region of Uckermark is facing a heavy decrease in groundwater level. This decrease is an indicator of forest drought in the region^49^.The forest drought can be hazardous for local biodiversity and slowly shrink down the size of existing surface water bodies. After the 2018 drought, central and northern Europe is facing adverse changes in its regional climate^50^.Being situated in the semi-arid region, Uckermark is one of the worst victims of this change. LSTM is a vital machine learning tool for short-term forecasting. But it is difficult to train LSTM and any type of RNN because it takes a lot of data to understand the pattern. In the case of this research groundwater level data of 20 years was used; where a recent study by Solgi et al^51^ used groundwater level data from December 1932 to July 2020 and another study by Wunsch et al.^52^ used groundwater level data from 1970 to 2015 In this study only seasonal peak data was used to represent the season. This technique was applied because the pattern of data collection for all the stations was not the same and thus all the data could not be used for inconsistency. One of the main aims of this study was to see whether using small time series is feasible for short-term forecasting; thus, a smaller time series (2000–2020) has been used. Though, the statistical tests have shown good results of the model’s accuracy, but still, if the inputs were more, the future predictions would be more precise. But in the case of natural phenomena like increase and decrease in the groundwater table, a lot of environmental and anthropogenic factors are involved. So, more complex models should be developed for more accurate predictions. If the groundwater measurement points were well distributed all around Uckermark, the decreasing trend would be more prominently visible. If the groundwater depletion and drought continue, the green abundance of Uckermark will start to decrease soon. The district or lower-level water authority can approve up to 2000 m$^3$ of water extraction per year, for greater extraction approval of upper authority is needed^53^.According to section 40, paragraph 4, No. 2 of the Brandenburg Water Act, a fee is charged if the rate of water withdrawal is more than 3000 m$^3$. The Policymakers and environmental agencies should take this phenomenon seriously and investigate the increase in seasonal depletion of groundwater table in this region.

## Software used

All the maps used in the papers were produced using ArcGIS Pro 2.8.3 by ESRI Inc. (https://www.arcgis.com). The licence for the softweare has been provided by the Hochschule für Technik Stuttgart in Germany.

## References

[1] Füssel, H. *et al.* Climate change, impacts and vulnerability in europe 2012. *An Indicator-Based Report. Luxembourg: Publ. Off. Eur. Union* (2012).

[2] Hellwig J, de Graaf I, Weiler M, Stahl K (2020). Large-scale assessment of delayed groundwater responses to drought. Water Resour. Res..

[3] Vogt, J., Niemeyer, S., Somma, F., Beaudin, I. & Viau, A. Drought monitoring from space. In *Drought and Drought Mitigation in Europe* 167–183 (Springer, 2000).

[4] Li, B. & Rodell, M. Groundwater drought: environmental controls and monitoring. In *Global Groundwater* 145–162 (Elsevier, 2021).

[5] Hughes J, Petrone K, Silberstein R (2012). Drought, groundwater storage and stream flow decline in southwestern Australia. Geophys. Res. Lett..

[6] Markonis Y (2021). The rise of compound warm-season droughts in Europe. Sci. Adv..

[7] Zhongming, Z., Wangqiang, Z. & Wei, L. Climate change, impacts and vulnerability in Europe 2012: An indicator-based report. *EEA Rep.* (2012).

[8] Rücker J, Nixdorf B, Quiel K, Grüneberg B (2019). North German lowland lakes miss ecological water quality standards-a lake type specific analysis. Water.

[9] Merz C, Steidl J (2015). Data on geochemical and hydraulic properties of a characteristic confined/unconfined aquifer system of the younger pleistocene in northeast Germany. Earth Syst. Sci. Data.

[10] Drastig K, Prochnow A, Baumecker M, Berg W, Brunsch R (2011). Agricultural Water Management in Brandenburg.

[11] Dalin C, Wada Y, Kastner T, Puma MJ (2017). Groundwater depletion embedded in international food trade. Nature.

[12] Richey AS (2015). Quantifying renewable groundwater stress with grace. Water Resour. Res..

[13] Lischeid G, Natkhin M (2011). The potential of land-use change to mitigate water scarcity in northeast Germany: A review. Die Erde.

[14] Merz C, Pekdeger A (2011). Anthropogenic changes in the landscape hydrology of the Berlin-Brandenburg region. Die Erde.

[15] Nowreen S (2021). Development of potential map for groundwater abstraction in the northwest region of Bangladesh using RS-GIS-based weighted overlay analysis and water-table-fluctuation technique. Environ. Monit. Assess..

[16] Al-Ruzouq R (2019). Potential groundwater zone mapping based on geo-hydrological considerations and multi-criteria spatial analysis: North UAE. Catena.

[17] Patra S, Mishra P, Mahapatra SC (2018). Delineation of groundwater potential zone for sustainable development: A case study from ganga alluvial plain covering Hooghly district of India using remote sensing, geographic information system and analytic hierarchy process. J. Clean. Prod..

[18] Nasir MJ, Khan S, Zahid H, Khan A (2018). Delineation of groundwater potential zones using GIS and multi influence factor (MIF) techniques: A study of district swat, Khyber Pakhtunkhwa,Ppakistan. Environ. Earth Sci..

[19] Selvam S, Magesh N, Chidambaram S, Rajamanickam M, Sashikkumar M (2015). A GIS based identification of groundwater recharge potential zones using RS and if technique: A case study in Ottapidaram taluk, Tuticorin district, Tamil Nadu. Environ. Earth Sci..

[20] Chen, C. *et al.**A Novel Deep Learning Algorithm for Groundwater Level Prediction Based on Spatiotemporal Attention Mechanism* (Res, Square(Pre Print), 2020).

[21] Hussein EA, Thron C, Ghaziasgar M, Bagula A, Vaccari M (2020). Groundwater prediction using machine-learning tools. Algorithms.

[22] Mukhopadhaya S, Kumar A, Stein A (2018). FCM approach of similarity and dissimilarity measures with $$\alpha $$-cut for handling mixed pixels. Remote Sens..

[23] Sun AY, Tang G (2016). Downscaling satellite and reanalysis precipitation products using attention-based deep convolutional neural nets. Broadening Use Mach. Learn. Hydrol..

[24] Pawul M, Śliwka M (2016). Application of artificial neural networks for prediction of air pollution levels in environmental monitoring. J. Ecol. Eng..

[25] Bowes BD, Sadler JM, Morsy MM, Behl M, Goodall JL (2019). Forecasting groundwater table in a flood prone coastal city with long short-term memory and recurrent neural networks. Water.

[26] Wunsch A, Liesch T, Broda S (2020). Groundwater level forecasting with artificial neural networks: A comparison of LSTM, CNN and NARX. Hydrol. Earth Syst. Sci. Discuss..

[27] Vyse SA, Taie Semiromi M, Lischeid G, Merz C (2020). Characterizing hydrological processes within kettle holes using stable water isotopes in the Uckermark of northern Brandenburg, Germany. Hydrol. Process..

[28] Ehlers J, Grube A, Stephan H-J, Wansa S (2011). Pleistocene glaciations of north Germany-new results. Dev. Quat. Sci..

[29] Atanasova-Pacemska, T., Lapevski, M. & Timovski, R. Analytical hierarchical process (AHP) method application in the process of selection and evaluation. *Int. Sci. Conf. Tech. Univ. Gabrovo* (2014).

[30] Dikmen Toker İ, Birgönül MT (2006). An analytic hierarchy process based model for risk and opportunity assessment of international construction projects. Can. Sci. Publ..

[31] Saaty TL (2019). What is the analytic hierarchy process?. Math. Models Decis. Support.

[32] Arulbalaji P, Padmalal D, Sreelash K (2019). GIS and AHP techniques based delineation of groundwater potential zones: A case study from southern western Ghats, India. Sci. Rep..

[33] Donnini M, Marchesini I, Zucchini A (2020). Geo-lim: A new geo-lithological map for central Europe (Germany, France, Switzerland, Austria, Slovenia, and Northern Italy) as a tool for the estimation of atmospheric CO2 consumption. J. Maps.

[34] Deepa S, Venkateswaran S, Ayyandurai R, Kannan R, Prabhu MV (2016). Groundwater recharge potential zones mapping in upper Manimuktha sub basin Vellar River Tamil Nadu India using GIS and remote sensing techniques. Model. Earth Syst. Environ..

[35] Magesh NS, Chandrasekar N, Soundranayagam JP (2012). Delineation of groundwater potential zones in Theni district, Tamil Nadu, using remote sensing, GIS and MIF techniques. Geosci. Front..

[36] Souissi D (2018). Mapping groundwater recharge potential zones in arid region using GIS and landsat approaches, Southeast Tunisia. Hydrol. Sci. J..

[37] Ferozur RM, Jahan CS, Arefin R, Mazumder QH (2019). Groundwater potentiality study in drought prone Barind tract, NW Bangladesh using remote sensing and GIS. Groundw. Sustain..

[38] Shaban A, Khawlie M, Abdallah C (2006). Use of remote sensing and GIS to determine recharge potential zones: The case of occidental Lebanon. Hydrogeol. J..

[39] Das S, Pardeshi SD (2018). Integration of different influencing factors in GIS to delineate groundwater potential areas using if and FR techniques: A study of Pravara basin, Maharashtra, India. Appl. Water Sci..

[40] Hochreiter S, Schmidhuber J (1997). Long short-term memory. Neural Comput..

[41] Shin M-J, Moon S-H, Kang KG, Moon D-C, Koh H-J (2020). Analysis of groundwater level variations caused by the changes in groundwater withdrawals using long short-term memory network. Hydrology.

[42] Wang Q, Kang K, Zhang Z, Cao D (2020). Application of LSTM and conv1d LSTM network in stock forecasting model. Artif. Intell. Adv..

[43] Nash JE, Sutcliffe JV (1970). River flow forecasting through conceptual models part i—A discussion of principles. J. Hydrol..

[44] Aye PP, Koontanakulvong S, Long TT (2017). Estimation of groundwater flow budget in the upper central plain, Thailand from regional groundwater model. SSMS. Jp.

[45] Andersen J, Refsgaard JC, Jensen KH (2001). Distributed hydrological modelling of the Senegal river basin-model construction and validation. J. Hydrol..

[46] Wulf M, Jahn U, Meier K (2016). Land cover composition determinants in the Uckermark (NE Germany) over a 220-year period. Reg. Environ. Chang..

[47] Gutzler C (2015). Agricultural land use changes—A scenario-based sustainability impact assessment for Brandenburg, Germany. Ecol. Indic..

[48] Mukhopadhaya S (2016). Rainfall mapping using ordinary kriging technique: Case study: Tunisia. J. Basic Appl. Eng..

[49] Mukhopadhaya S (2016). Rainfall mapping using ordinary kriging technique: Case study: Tunisia. J. Basic Appl. Eng. Res..

[50] Ramonet M (2020). The fingerprint of the summer 2018 drought in Europe on ground-based atmospheric CO2 measurements. Philos. Trans. R. Soc..

[51] Solgi E, Jalili M (2021). Zoning and human health risk assessment of arsenic and nitrate contamination in groundwater of agricultural areas of the twenty two village with geostatistics (case study: Chahardoli plain of Gorveh, Kurdistan province, Iran). Agric. Water Manag..

[52] Wunsch A, Liesch T, Broda S (2021). Groundwater level forecasting with artificial neural networks: A comparison of long short-term memory (LSTM), convolutional neural networks (CNNS), and non-linear autoregressive networks with exogenous input (NARX). Hydrol. Earth Syst. Sci..

[53] Brandenburg, L. Environmental data brandenburg 2008/09. *Brandenburg State Off. for Environ. (LUA), Potsdam* 130 (2009).

